# The Physiopathology of T- Cell Acute Lymphoblastic Leukemia: Focus on Molecular Aspects

**DOI:** 10.3389/fonc.2020.00273

**Published:** 2020-02-28

**Authors:** Bruno Fattizzo, Jessica Rosa, Juri Alessandro Giannotta, Luca Baldini, Nicola Stefano Fracchiolla

**Affiliations:** ^1^Fondazione IRCCS Ca' Granda Ospedale Maggiore Policlinico di Milano, Milan, Italy; ^2^Dipartimento di Oncologia ed Oncoematologia, Università degli studi di Milano, Milan, Italy

**Keywords:** T-cell acute lymphoblastic leukemia, genome, molecular, target therapies, early T cell precursors acute lymphoblastic leukemia

## Abstract

T-cell acute lymphoblastic leukemia/lymphoma is an aggressive hematological neoplasm whose classification is still based on immunophenotypic findings. Frontline treatment encompass high intensity combination chemotherapy with good overall survival; however, relapsing/refractory patients have very limited options. In the last years, the understanding of molecular physiopathology of this disease, lead to the identification of a subset of patients with peculiar genetic profile, namely “early T-cell precursors” lymphoblastic leukemia, characterized by dismal outcome and indication to frontline allogeneic bone marrow transplant. In general, the most common mutations occur in the NOTCH1/FBXW7 pathway (60% of adult patients), with a positive prognostic impact. Other pathogenic steps encompass transcriptional deregulation of oncogenes/oncosuppressors, cell cycle deregulation, kinase signaling (including IL7R-JAK-STAT pathway, PI3K/AKT/mTOR pathway, RAS/MAPK signaling pathway, ABL1 signaling pathway), epigenetic deregulation, ribosomal dysfunction, and altered expression of oncogenic miRNAs or long non-coding RNA. The insight in the genomic landscape of the disease paves the way to the use of novel targeted drugs that might improve the outcome, particularly in relapse/refractory patients. In this review, we analyse available literature on T-ALL pathogenesis, focusing on molecular aspects of clinical, prognostic, and therapeutic significance.

## Introduction

T-cell acute lymphoblastic leukemia/lymphoma (T-ALL/-LL) is an aggressive hematological tumor, driven by malignant transformation and expansion of T-cell progenitors. T-ALL and T-LL are distinguished by the presence of more or <20% marrow blasts, respectively (1, 2). The *2016 revision* of *WHO classification* added a provisional entity called *Early T-cell precursor* (ETP) *ALL*. This subset is characterized by a unique immunophenotypic (reduced expression of T-cell markers, CD1a, CD8, and CD5) and genetic profile, indicating only limited early T-cell differentiation, with retention of some myeloid and stem cell characteristics (2).

Current treatment of T-ALL consists of high intensity combination chemotherapy, resulting in high overall survival, with the best outcomes observed in pediatric patients (3). Despite the high response rates after first-line therapy, about 20% of pediatric and 40% of adult patients will relapse (4). Differently from B-cell precursors ALL, where highly effective monoclonal antibodies as well as CD19 targeting chimeric antigen receptor (CAR) T-cells have been developed, in T-ALL only the purine nucleoside analog nelarabine is licensed for relapsed/refractory patients (1, 5). Relapsed/ refractory T-ALL treatment is therefore an unmet need and only new targeted drugs will have the potential to overturn the outcome of these patients.

The purpose of this review is to analyse available data on T-ALL pathogenesis, starting with a brief description of current T-ALL classification and treatment, and then focusing on molecular aspects of clinical, prognostic, and therapeutic significance.

## Results

### Snapshot on T-ALL Diagnosis, Classification, and Therapy

Diagnosis of T-cell ALL relies on a combination of morphology, immunophenotype, and cytogenetic features, many of which inform prognosis and treatment choices. The morphological distinction between L1 and L2 blasts has now lost clinical relevance since more precise immunophenotypic categories have been set. One of the most widely used is the European Group for the Immunological Characterization of Leukaemias subclassification based on the various stages of T-cell maturation (6). T-lymphoblasts are TdT+ and show positivity for cytoplasmic CD3, the only lineage specific marker. The variable expression of CD1a, CD2, CD4, CD5, CD7, and CD8 distinguishes pro-, pre-, cortical, and mature T-ALL. As regards the relationship between immunophenotype and prognosis, the best outcomes have been observed in the cortical T-cell ALL, while CD1a-negative patients show an increased relapse rate and a lower survival (7, 8). Noteworthy, ETP-ALL is a novel subcategory of T-ALL, characterized by a distinct gene expression profile and immunophenotype. ETP-ALL cells are tipically CD7+ but CD1a– and CD8–, CD5 weak, and express >1 myeloid or stem cell marker (i.e., CD34, CD13, or CD33). These cells originate from a subset of immature thymocytes directly derived from hematopoietic stem cells, thus able to differentiate into both T- and myeloid cells. ETP-ALL accounts for 15% of all T-cell ALL in children and about 35% in adult T-cell disease (9, 10).

As occurs in B-cell ALL, also in T-cell ALL prognosis is influenced by cytogenetics. In a large trial cytogenetic analysis displayed an abnormal karyotype in 72% of patients, with complex karyotypes (≥5 abnormalities) in about 8% of cases, significantly impacting on prognosis (5-year OS 19 vs. 51%, *p* = 0.006) (11). An increasing number of molecular abnormalities have been associated with T-cell ALL and will be discussed in a dedicated paragraph.

### First Therapy Line

Regarding therapy, in the first-line setting, the standard of care for fit patients consists of ALL-based pediatric-inspired regimens, incorporating induction (combination of steroids, anthracyclines, and vincristine), consolidation, delayed intensification, and maintenance with central nervous system (CNS) prophylaxis (12, 13). Addition of the enzyme l-asparaginase, and more recently its pegylated *E. coli*-derived form (PEG-ASP), characterized by longer half-life and less anti-drug antibody formation, has been demonstrated to significantly improve response rates and OS both in pediatric (14) and adult patients (15, 16). As occurs in B-cell ALL, indication to allogenic hematopoietic stem cell transplant (alloHSCT) in T-ALL in first remission is based on high risk features at diagnosis and is more and more frequently MRD-driven (17). CNS involvement at diagnosis is more likely in T- than in B-cell ALL (9.6 vs. 4.4%; *p* = 0.001) and has been associated with inferior 5-year OS due to an increased risk of both systemic and CNS relapse (18). The most common prophylaxis employed is the combination of high-dose IV methotrexate and intrathecal chemotherapy (7, 11). A randomized trial stressed the importance of the use of 5 g/sq.m. in T-ALL, higher than those used in B-cell ALL (19). As regards ETP-ALL, a Spanish multicentre study showed the worse prognosis to be ascribed to a lower response to induction therapy than to an increased relapse rate, suggesting that use of different schedules, such as fludarabine, cytarabine, G-CSF, idarubicin (FLAG-IDA), and other more myeloid-oriented chemotherapies, or FLT3-targeted therapies, may play an advantage in this subcategory of patients (20). Current consolidation strategies comprise a delayed intensification including drugs used in induction phase, followed by a 2-year maintenance with 6-mercaptopurine and methotrexate, pulses of vincristine and steroids, and additional IT CNS prophylaxis. Molecular-based and flow cytometry-based techniques allow reliable assessment of minimal residual disease (MRD), whose monitoring at precise timepoints is the standard of care for ALL patients treated with curative intent. The molecular method consists of identifying clone-specific rearrangement with Sanger on next-generation sequencing into the immunoglobulin heavy chain gene or T-cell receptor genes by using a large panel of consensus primers, generating patient-specific real-time quantitative polymerase chain reaction assays for quantification in about 90% of cases, with a quantitative range of 10^−4^. Despite variable definitions of “early” assessment of MRD (from 6 to 10–16 weeks from the start of therapy), plenty of studies in ALL have confirmed that early MRD response is the most powerful predictor of long-term survival in adult patients with ALL (21–23). Finally, myeloablative alloHSCT should be considered for high-risk T-cell disease. Allocation to alloHSCT may vary among study groups, but generally speaking, failure to achieve CR after induction therapy, high white cell count at presentation, high risk cytogenetics/immunophenotype, and MRD persistence at defined timepoints can all be used to allocate to transplant (11, 24, 25). As regards the subcategory of ETP-ALL, two trials demonstrated improvement in survival in ETP-ALL patients transplanted early in case of treatment resistance (20). Considered its better prognosis, consolidation with alloHSCT is not considered necessary in T-LBL, unless suggested by an adverse course of the disease (26).

### Relapsed Disease

About 80% of relapses occur within 2 years of diagnosis. With <7% of survival rate at 5 years (27), relapsed T-ALL has dismal outcome, and no standard strategies are available so far. Response rates using standard chemotherapy regimens such as FLAG-IDA are around 30–40%, with a median OS of 6 months in responders (28). Nelarabine is the only new agent specifically licensed for relapsed/refractory T-cell ALL/LBL. Used as single agent, this drug induced ORR of 14–55% in pediatric patients (29) and 41–46% in adults, with 1-year OS of 28% (30). Neurotoxicity is the major toxicity, affecting around 15% of patients, with more severe and irreversible cases in a minority of patients (31). Importantly, most of the patients obtaining a CR with nelarabine were able to proceed to alloHSCT.

### Focus on the Molecular Pathways Involved in T-ALL Pathophysiology

**Figure 1:**
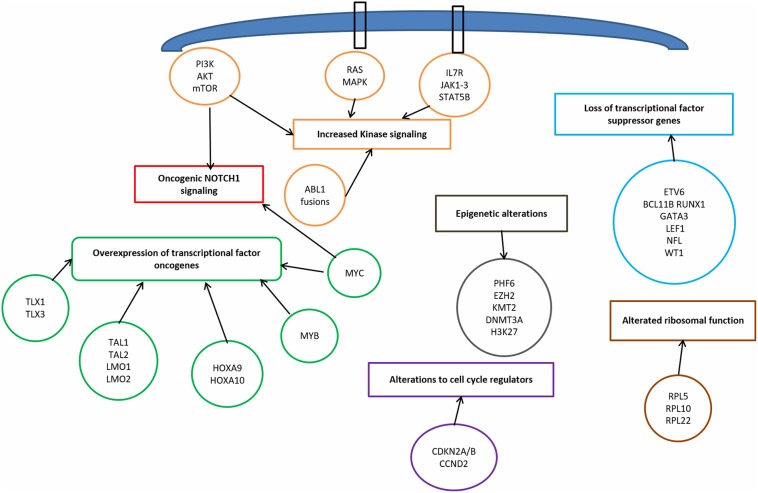
Signaling pathways involved in T-cell acute lymphoblastic leukemia pathophysiology.

T-ALL results from a multistep transformation process in which the accumulation of genetic alterations affects key oncogenic/tumor suppressors pathways, that are responsible for proliferation, survival and differentiation of T-cells (32, 33). The molecular steps involved in T-ALL pathogenesis encompass: transcriptional deregulation of oncogenes/oncosuppressors, NOTCH1 signaling, cell cycle deregulation, kinase signaling (including IL7R-JAK-STAT pathway, PI3K/AKT/mTOR pathway, RAS/MAPK signaling pathway, ABL1 signaling pathway), epigenetic deregulation, ribosomal dysfunction, and altered expression of oncogenic miRNAs or long non coding RNA (34) (Figure 1).

### Transcriptional Deregulation of Oncogenes and Oncosoppressors

Among the genetic abnormalities, chromosomal translocations of transcription factor oncogenes to regulatory regions of T-cell receptor (TCR) genes are characteristic of T-ALL (34). Approximately 50% of patients harbor chromosomal translocations involving 14q11 (TCR alfa and TCR delta) and 7q34 (TCR beta) (35). Other mechanisms involved are chromosomal rearrangements with other regulatory sequences, duplication/amplification, and mutations or small insertions generating novel regulatory sequences acting as enhancers (36).

Transcriptional factors belonging to bHLH, LMO, and HOX families are also implicated (Table 1). The largest subgroup, representing about 30–35% of T-ALL, is characterized by the abnormal expression of TAL1 (1p32), a bHLH member, which results from either *t*_(1;14)_ (p32;q11), and *t*_(1;7)_ (p32;q35) translocations, small insertions, mutations or 1p32 deletion (36, 37). TAL1 expression is associated with a late cortical thymocyte immunophenotype (CD1a-) (38), and correlates with favorable outcomes (35, 39).

**Table 1:** Molecular pathways involved in T-ALL pathogenesis.

**Gene**	**Locus**	**Type of mutation**	**Frequency**	**Relevance**	**References**
**TRANSCRIPTION REGULATOR (ONCOGENE)**					
TAL1	1p32	Aberrant expression due to translocations involving one of the TCR gene [TCRalfa (14q11) or TCRbeta (7q34)]; duplications or amplifications; mutations or insertions;	30–35%	Favorable outcome	(35–39) (40, 41)
TAL2	10q24		Rare	–	
TLX1/HOX11	10q24		5–10% (children), 30% (adults)	Favorable outcome	(32, 35, 38, 39, 42–47)
TLX3/HOX11L2	5q35		20–25% (children), 5% (adults)	Poor outcome	
LMO1	11p15	Aberrant expression due to t (11,14) or small deletion	15%	Favorable outcome	(32, 35, 45, 48, 49)
LMO2	11p13				
HOXA9;HOXA10	7p15	Chromosomal translocations and inversions involving TCRs loci	3%	–	(32)
NKX2-1;NKX2-2	14q13; 20p11		5% (children)	–	
MLL	11q23	Rearrangements with various partners	5% (children)	Poor outcome	(33)
MYC	8q24	Mutations or rearrangements or amplifications, rarely t (8,14)(q24;q11)/MYC-TCRalfa	6%	Subclonal; poor outcome; more common in T-LL	(32, 50–54)
MYB	6q23		10%	–	(45, 55)
**TRANSCRIPTION REGULATOR (ONCOSOPPRESSOR)**					
BCL11B	14q3	Deletions or inactivating mutations	10%	–	(32, 56–60)
ETV6^*^	12p13		13% (25% of ETP)	In etp, poor outcome	
RUNX1^*^	21q22		10–20% (most in ETP)	In ETP, poor outcome	
GATA3^*^	10p14		5% (most in ETP)	In ETP, poor outcome	
LEF1	4q24		10–15%	IF early T cortical	
WT1	11p13		10%	–	
NF1^*^	17q11		More common in children	–	
**NOTCH1 SIGNALING**					
NOTCH1	9q34.3	activating mutations most of all; t (7,9)(q34;q34)/TCRbeta-NOTCH1 in <1% of cases	60–70%	Favorable outcome; NOTCH inhitors	(33, 51, 61–69)
FBXW7	4q31.3	Loss of function mutations	15%	Prognostic if evaluated in combination with NOTCH1	
**CELL CYCLE REGULATION**					
CDKN2A (p16INK4A; p14ARF); CDKN2B (p15)	9p21	Deletions	70%	–	(32, 70, 71)
CDKN1B (p27KIP1)	12p13	Deletions	12%	–	
CCND2(cyclin D2)	12p13	Chromosomal translocations involving TCRs loci	3%	–	
RB1	13q14	Deletions	15%	–	
**IL7-JAK-STAT PATHWAY**					
IL7R^*^	5p13	Activating mutation	20–30% (most in ETP): JAK3 16%; JAK1 10%; IL7R 10%; STAT5B 5–10%	–	(33, 34, 36, 72–75)
JAK1^*^	1p32.3-p31.3	Gain of function mutations		poor outcome; JAK inhibitors	
JAK2	12p13	Translocation t (9,12)(p24;p13) involving ETV6-JAK2		–	
JAK3^*^	19p13-p12	Gain of function mutations		in ETP, poor outcome	
STAT5B	17q21.2	Gain of function mutations		–	
DNM2	19p13.2	Loss-of-function		–	(76)
PTPN2	18p11.3-p11.2	Inactivating mutations	6%	–	
PTPRC (CD45)	1q31.3-q32.1	Inactivating mutations		–	
PIM1	6p21	t (6, 7)(p21; q34)/PIM1-TCRbeta	5%	–	
**PI3K-AKT-mTOR PATHWAY**					
PI3K	3q26	Gain of function mutations	5%	PI3K inhibitors	(77–80)
AKT	14q32	Gain of function mutations	2%	–	
PTEN	10q23	Loss of function mutations, deletions	10-15%	–	
mTOR	1p36.22	Gain of function mutations	<1%	mTOR inhibitors	
**RAS PATHWAY**					
RAS (N-RAS, K-RAS, H-RAS)^*^	1p13; 12p12; 11p15	Activating mutations	Most in ETP	Poor outcome	(34, 35, 66, 74, 81)
NF1^*^, PTPN11	17q11; 12q22	Loss of function mutations	Most in ETP	in ETP, poor outcome	(82, 83)
**ABL KINASE SIGNALING**					
ABL1	9q34	Rearrangements, episomal amplifications (NUP214-ABL1; EML1-ABL; ETV6-ABL)	8%	TK inhibitors	(35, 84–86)
**EPIGENETIC REGULATION**					
PHF6	Xq26	Inactivating mutations or deletions	16% (children), 38% (adults), M>>>F	–	(36, 87)
KDM6A	Xp11		6–7%	–	
EZH2^*^ (and others of PCR2 complex)	7q36		25%	in ETP, poor outcome	
DNMT3A^*^	2p23		15% (adults), most in ETP	in ETP, poor outcome	
H3K27	1q42			–	
**RIBOSOMAL FUNCTION**					
RPL5	1p22	Inactivating mutations	2%	–	(32, 88)
RPL10	Xq28	Missense mutations at residue R98	6–8% (children)	Hypoproliferative phenotype	
RPL11	1p36	Inactivating mutations	1%	-	

* *Genes more commonly involved in ETP-ALL. TK, tyrosine kinase; ETP, early T-cell precursor*.

LMO1 (11p15) and LMO2 (11p13) are part of a transcriptional complex, and are aberrantly expressed at high levels in ~15% of T-ALL, due to both translocations to TCR loci and small chromosomal deletions (32, 45, 48, 49). Also these cases carry a favorable prognosis (35).

Among HOX genes family, TLX1 (10q24, formerly HOX11), and TLX3 (5q35) are over-expressed in T-ALL. TLX1+T-ALLs represent 30% of adult T-ALLs and result from the translocation *t*_(10;14)_ (q24,q11); the latter involves the TCR locus (42, 43) and contributes to thymocyte arrest at the early cortical stage (CD1a+), conferring favorable outcome (32, 44, 45). On the contrary, TLX3 overexpression (20–25% of pediatric T-ALL) correlates with a poor outcome; it results from *t*_(5;14)_ which places this oncogene under the control of T-cell regulatory sequences in the BCL11B locus (32, 35, 39, 46, 47).

### Other Protoncogenes Involved: MLL, MYC, and MYB

MLL (11q23), originally described in pediatric acute myeloid leukemia, is also involved in T-ALL pathogenesis. The outcome of MLL-rearranged leukemias is generally unfavorable, however this relationship is less clear in T-ALL. MLL-MLLT1 rearrangement, present in 2–3% of T-ALL, has a better outcome, whereas PICALM-MLLT10 rearrangement (about 6–7% of cases) is linked to worse prognosis (39, 89, 90).

MYC (8q24) and MYB (6q23) are proto-oncogenes involved in the transcriptional deregulation observed in T-ALL. In early T-cell development, MYC plays an important role in the control of cell growth downstream NOTCH1 and TCR signaling (50). Moreover, rearrangements involving PI3K/AKT pathway often result in MYC overexpression (52). The translocation *t*_(8;14)_, involving the TCR, is present in only 1% of MYC+ T-ALL (53), and other mechanisms occur: translocations involving others partners, duplications, amplifications, and reduced degradation (32). In a subgroup of about 6% of T-ALL, MYC translocations are secondary abnormalities, present in subclones, and are associated with induction failure, high rate of relapse, and with an aggressive clinical course (52). The genetic profile of these MYC- translocated T-ALL is characterized by concomitant abnormalities, including CDKN2A/B deletions, PTEN inactivation, and mutations typical of myeloid neoplasms, such as DNMT3A (54). Regarding MYB, it is activated in T-ALL harboring the *t*_(6;7)_ translocation, which is common among children younger than 2 years of age, or as a result of duplications or amplification of 6q23 (45, 55).

In addition to oncogenes, tumor suppressors contribute to transcriptional deregulation in T-ALL, usually due to deletions or inactivating mutations. BCL11B (14q32), ETV6 (12p13), RUNX1 (21q22), GATA3 (10p14), LEF1 (4q24), WT1 (11p13), and NF1 (17q11) are the main oncosoppressors involved (32).

ETV6, RUNX1, and GATA3, described also in acute myeloid leukemia, are deleted or inactivated in ETP-ALL, and correlate with poor outcome: ETV6 (12p13) mutations account for ~25% of ETP-ALL (56), whilst RUNX1 (21q22), and GATA3 (10p14) mutations are less common. BCL11B (14q32) is mutated in 10% of T-ALL (57); LEF1 (4q24) in 10–15% and is associated with an early cortical thymocyte immunophenotype (58), and WT1 (11p13) in about 10% of cases (59). Monoallelic deletion of 17q12, involving the tumor suppressor NF1, is common in children, but it has been described also in adults (60).

### NOTCH1 Pathway

NOTCH1 pathway is essential for T-cell lineage commitment and maturation of hematopoietic progenitors (61). Rarely, the *t*_(7;9)_ (q34;q34.3) translocation leads to the expression of a constitutively active form of NOTCH1 (9q34.3) (62). However, in over 60% of T-ALLs, NOTCH1 aberrant expression results from activating mutations (63). These mutations lead to ligand-independent cleavage and activation of the intracellular NOTCH1 domain and to the stabilization of the active protein (33). Loss of function of negative regulators of NOTCH1 is an alternative mechanism. As a matter of fact, 10–15% of T-ALL, harbor mutations in FBXW7 (4q31.3), a protein that promotes NOTCH1 proteasomal degradation, and lead to increased NOTCH1 protein stability (64). In prognostic models, patients with NOTCH1 and FBXW7 mutations are defined as low risk cases (65, 66).

NOTCH1 pathway is also a central driver of T-cell metabolism and promotes leukemia cell growth via direct upregulation of anabolic pathways, including ribosome biosynthesis, protein translation and nucleotide and aminoacid metabolism. The effect on cell growth is enhanced by the upregulation of MYC (51, 67, 68). Furthermore, NOTCH1 activates mTOR/Akt pathway and increases the glucose uptake in maturating thymocytes. In summary, oncogenic Notch1 pathway is responsible for enhanced aerobic glycolysis and upregulation of anabolic pathway leading to increased proliferation (69).

### Cell Cycle Deregulation

The loss of cell cycle control has a prominent role in the pathogenesis of T-ALL. Deletions of the cyclin-dependent kinase inhibitor 2A (CDKN2A encoding tumor suppressors p16^INK4A^ and p14^ARF^) and 2B (CDKN2B encoding the tumor suppressor p15^INK4B^) loci on 9p21 are present in up to 70% of T-ALL, leading to abnormal proliferation control (70). Moreover, deletions in retinoblastoma 1 (RB1, locus on 13q14), a regulator of cell cycle progression, are found in 15% of T-ALL, and deletions involving the CDKN1B locus (12p13, encoding p27^KIP1^) are present in about 12% loci (32). Finally, high levels of cyclin D (CCND2) are present in 3% of T-ALLs, as a result of translocations with TCR loci (71).

### Kinase Signaling Pathways

Kinase signaling pathways aberrantly activated in T-ALL include IL7R/JAK/STAT, PI3K/AKT/mTOR, RAS/MAPK, and ABL kinase signaling (34, 36).

IL7R/JAK/STAT pathway is essential for normal T-cell development and is triggered by the interaction between IL7 and its heterodimeric receptor. Upon ligand-binding, IL7R dimerizes and induces JAK1 and JAK3 phosphorylation, with consequent STAT5 activation. STAT5 dimerizes and translocates to the nucleus, where regulates many target genes, including BCL2 family members (72, 73). Activating mutations of IL7R (5p13), JAK1 (1p32), JAK3 (19p13), and/or STAT5B (17q21) are present in 20–30% of T-ALL cases, with a higher frequency in ETP-ALL patients (33, 74). JAK3 mutations are present in about 16% of T-ALL cases, and a strong association between JAK3 mutations and HOXA9 expression has been demonstrated (75). Furthermore, 6% of T-ALLs are characterized by haplo-insufficiency of negative regulators of this pathway, such as DNM2 (19p13), PTPN2 (18p11), and PTPRC (1q31) (76). The rare *t*_(9,12)_ (p24;p13) translocation encodes a constitutively active kinase protein, ETV-JAK2, leading to aberrant JAK signaling (91). PIM1 is the ultimate target of the JAK/STAT downstream, and high PIM1 expression is a biomarker of activation of this pathway; PIM1 can be overexpressed also as a result of translocation *t*_(6,7)_ (p21;q34), involving TCR beta (76).

PI3K/AKT/mTOR pathway is aberrantly activated in T-ALL, resulting in enhanced cell metabolism, proliferation, survival, differentiation, and impaired apoptosis (77). Hyperactivation of this oncogenic pathway is mainly caused by loss-of-function mutations/deletions of PTEN (10q23), occurring in about 10–15% of T-ALLs (78, 79). Additional mutations include gain-of-function mutations in regulatory and catalytic subunits of PI3K (3q26) (4,5% of cases), or in AKT (14q32) or mTOR (1p36) (2 and <1% of cases, respectively) (80).

RAS proteins, including H-RAS (11p15), N-RAS (1p13), and K-RAS (12p12), are fundamental signal transductors from cell surface to downstream effectors (34). RAS-MAPK signaling pathway is frequently hyperactivated in T-ALL, and RAS mutations are present in about 5–10% of cases, particularly in high risk ETP-ALL and in relapsing patients (35, 66, 74, 81). RAS pathway regulators may also be mutated: loss-of-function of NF1 (17q11) and PTPN11 (12q22) have been described in 3% of cases (82, 83).

Finally, ABL1 gene (9q34) is rearranged in 8% of cases, leading to constitutive kinase activity (84). The most frequent rearrangement is NUP214-ABL1 amplification (9q34 amplification), observed in 6% of patients (85), whilst EML1-ABL and ETV6-ABL1 are less common (35). NUP214-ABL is a secondary, subclonal alteration and has not been linked with poor prognosis (86).

### Epigenetic Deregulation

Mutations in epigenetic factors are frequent in T-ALL: PHF6 (Xq26), SUZ12 (17q11), EZH2 (7q36), TET2 (4q24), H3F3A (1q42), KDM6A (Xp11), EED (11q14), SETD2 (3p21), and DNMT3A (2p23) mutations are the most common (32, 35). Considering the most frequent, PHF6 is a histone modifier, involved in transcriptional regulation, DNA damage response and cell cycle control. Loss-of-function mutations or deletions of this gene, exclusively found in male patients, are present in 16% of pediatrics and 38% of adults, and result in G2/M cell cycle arrest. Mutational loss of PHF6 is associated with the aberrant expression of the transcription factor oncogenes TLX1 and TLX3 (87). H3K27 regulates methylation, and together with the PRC2 complex (polycomb repressive complex 2, that includes EZH2, SUZ12, and EED) is mutated in up to 25% of T-ALLs (36).

### Ribosomal Function

Ribosomes are cellular components required for protein synthesis, a crucial step in rapidly dividing leukemic cells. Ribosomal genes RPL5 (1p22), RPL10 (Xq28), and RPL11 (1p36) have been described to be mutated in T-ALL (32). RPL10 mutations are found in 6–8% of pediatrics, with the recurrent RPL10^R98S^ mutant allele in most cases (32, 88). RPL10^R98S^ mutant leukemia cells may increase the expression of anti-apoptotic protein BCL2. RPL10 R98S mutations are mutually exclusive with JAK/STAT mutations and are associated with a hypoproliferative phenotype (88).

### Novel Therapeutic Strategies

Regarding therapy, T-ALL is an aggressive leukemia with limited options, particularly in the relapsed/refractory setting. A better understanding of T-ALL pathogenesis may allow the development of molecular targeted therapies (Table 2) (49). For instance, the high prevalence and prominent role of NOTCH1 mutations make it a promising therapeutic target. Clinical trials have explored the use of γ-secretase inhibitors (86), with limited efficacy and gastrointestinal toxicity (92) that can be reduced by the addition of steroids (93, 104). An example is PF-03084014 that has been tested in a clinical study of relapsed/refractory T-ALL/T-LL (A8641014), with one out of 8 patients experiencing complete response lasting about 3 months (94). Other options are NOTCH transcriptional complex inhibitors or antibodies against NOCTH1 (105). Cell cycle dysregulation by CDK4/CDK6 altered pathway is another potential target, and CDK4/CDK6 inhibitors (86) such as palbociclib recently entered clinical trials. The constitutive activation of PI3K/AKT/mTOR signaling pathway may also be targeted: several PI3K inhibitors showed anti-leukemic effects in T-ALL cell lines, whereas mTOR inhibitors seem to prolong survival in T-ALL cells (34). The most studied molecules were everolimus and temsirolimus (106), that induced variable responses (0–50%) in association to chemotherapy and in a small number of cases (94–96). The limited efficacy of mTOR inhibitors seems to be linked to the activation of compensatory signaling pathways (106). Furthermore, dual PI3K/mTOR inhibitors, NVP-BEZ235 and NVP-BKM120, have been studied. BEZ235 had antiproliferative and proapoptotic effect in T-ALL cell lines (97), and a clinical trial has been started (NCT01756118). BKM120/Buparlisib showed modest efficacy and was tolerable in advanced acute leukemia (only 1 patient with T-ALL) in a recent clinical trial (98). As regards cytokine signaling, JAK-STAT pathway is activated in T-ALL and about 5% of cases are driven by tyrosine kinase oncogene fusions, particularly the NUP214-ABL1 rearrangement (86). JAK inhibitors, such as Ruxolitinib and Tofacitinib, have been studied in preclinical models with activation of IL7R/JAK/STAT pathway (34, 86). In addition, imatinib, dasatinib, and nilotinib are all active against *NUP214-ABL1*-positive T-cells, with different ability to inhibit this kinase and induce apoptosis in preclinical studies (102). Finally, RPL10^R98S^ mutant leukemia cells are potentially sensitive to Bcl2 inhibitor venetoclax (88). Venetoclax combined to chemotherapy induced a morphological remission in 60% of patients (including ETP-ALL) in a recent retrospective study (103).

**Table 2:** Clinical and preclinical trials with target therapies in T-cell acute lymphoblastic leukemia.

**Type of study**	**Molecule**	**Reference**
**NOTCH1 INHIBITORS**		
Clinical, phase 1	MK-0752	(92)
Preclinical	PF-03084014 + DEX	(93)
Clinical	PF-03084014	(94)
Clinical, phase 1b/2	Crenigacestat (LY3039478) + Dex	NCT02518113
Clinical, phase 1	BMS-906024 alone or + DEX	NCT01363817
Clinical, phase 1	BMS-906024	(95)
Clinical, phase 1	MK0752-013	NCT00100152
Clinical, phase 1/2	RO4929097	NCT01088763
**CDK4/6 INHIBITORS**		
Preclinical	LEE011 + a panel of drugs	(96)
Clinical, phase 1	Palbociclib + CT	NCT03792256/ AINV18P1
**PI3K/mTOR DUAL INHIBITORS**		
Preclinical	NVP-BEZ325/ Dactolisib	(97)
Clinical, phase 1	NVP-BEZ325/ Dactolisib	NCT01756118
Clinical, phase 1	NVP-BKM120	(98)
**mTOR INHIBITORS**		
Clinical, phase 1	Everolimus (RAD001) + CT	NCT01523977
Clinical, phase I/II	Everolimus + HyperCVAD	(99)
Clinical, phase I	Temsirolimus (CCI799) + UK ALL R3 (Dex+Mitox+VCR+pegAsp)	(100)
Clinical, phase I	Everolimus + CT (VCR, PDN, peg Asp, Doxo)	(101)
Clinical, phase II	Sapanisertib	NCT02484430
Clinical, phase 1	Sirolimus + HyperCVAD	NCT01184885
Clinical, phase 1	Temsirolimus + VP16 + CTX+ DEX	NCT01614197
Clinical, phase 1	Everolimus + Nelarabina+ CTX+ VP16	NCT03328104
**TK INHIBITORS**		
Clinical, phase 1/2	Ruxolitinib (doses ranging from 10–80 mg) + L-ASP, VCR, and PDN	NCT03613428
Preclinical	Imatinib or Dasatinib or Nilotinib	(102)
Clinical	Imatinib + CT	NCT00049569
**HDAC INHIBITORS (EPIGENETIC REGULATORS)**		
Clinical	Chidamide + CT	NCT03564704
	BCL2 inhibitors	
Clinical	Venetoclax + CT	(103)
Clinical, phase 1/2	Venetoclax + low intensity CT	NCT03808610
Clinical, phase 1b/2	Venetoclax + Vincristine	NCT03504644

*CT, chemotherapy; Dex, dexamethasone; VCR, vincristine, Mitox, mitoxantrone; Asp, asparaginase; Doxo, doxorubicine; VP16, etoposide; CTX, cyclophosphamide; PDN, prednisone; TK, tyrosine kinase; HDAC, histone deacetylase*.

## Discussion and Future Perspectives

T-ALL is a genetically heterogeneous disease caused by a multistep process, involving cell growth, proliferation and differentiation of T-cells (36, 66). A better understanding of the molecular physiopathology may refine classification and prognostication. Regarding the former, molecular findings allowed the definition of the ETP-ALL subgroup, characterized by a distinct gene expression profile and immunophenotype (9). Moreover, high frequencies of FLT3, NRAS/KRAS, DNMT3A, IDH1, and IDH2 mutations have been found in ETP-ALL (107), similarly to what observed in myeloid leukemic stem cells. This new entity is associated with high levels of minimal residual disease after induction chemotherapy (10) and inferior long-term outcomes (25, 108). Beyond ETP-ALL, other recurrent mutations carry prognostic significance. Among them, the most common occur in the NOTCH1/FBXW7 pathway (60% of adult patients) (63), and confer a positive prognosis in most studies (65, 109, 110). A risk classification based on the presence or absence of NOTCH1/FBXW7, PTEN, or N/K-RAS mutations has been proposed (111). The good-risk group (significantly superior OS and inferior cumulative incidence of relapse) harbored mutations in the NOTCH1/FBXW7 pathway with no associated mutations in PTEN or N/K-RAS; mutated NOTCH1/FBXW7 genes plus mutations in PTEN or N/K-RAS were classified as poor risk with OS 44% and cumulative incidence of relapse 54%.

The study of genetic lesions involved in T-ALL pathogenesis may lead to the development of new targeting drugs. In particular, different inhibitors of NOTCH1 pathway are under active study, including γ-secretase inhibitors, blocking of NOTCH transcriptional complex, and antibodies against NOCTH1. Cell cycle blockers like palbociclib and PI3K-, mTOR- and dual inhibitors (everolimus and temsirolimus, NVP-BEZ235 and NVP-BKM120), showed promising anti-leukemic effect both *in vitro* and *in vivo*. Tyrosine kinase inhibitors targeting IL7R/JAK/STAT pathway (ruxolitinib and tofacitinib) and NUP214-ABL1-mutated ALL (imatinib, dasatinib and nilotinib) are all active against T-cell blasts. Finally, Bcl2 inhibitor venetoclax may have a role in RPL10^R98S^ mutant ALL.

In conclusion, in the last years the better understanding of genetic lesions in T-ALL paved the way to novel target therapies, and many preclinical and clinical trials are ongoing. However, the rarity of the disease makes it hard to design specific trials, and the complexity of the molecular landscape may account for the limited efficacy of selective inhibitors in clinical studies. In this setting, differently from other leukemic contexts where chemo-free regimens are emerging (as observed for Ph+ B-ALL targeted with TK-inhibitors and bispecific antibodies), combination chemotherapy is still needed to establish a response. Nevertheless, the inhibition of more ancillary targets like Bcl2 seems to evoke better anti-leukemic effect and may lead the way for future studies and combinations.

## Author Contributions

BF, JR, and JG wrote the paper and revised it for intellectual content. NF designed the study and revised the paper for intellectual content. LB revised the manuscript for intellectual content. All authors made substantial contributions to the conception or design of the work, revised it critically for important intellectual content provided approval for publication of the content, and agree to be accountable for all aspects of the work in ensuring that questions related to the accuracy or integrity of any part of the work are appropriately investigated and resolved.

### Conflict of Interest

The authors declare that the research was conducted in the absence of any commercial or financial relationships that could be construed as a potential conflict of interest.
